# Conducting Developmental Research Online vs. In-Person: A Meta-Analysis

**DOI:** 10.1162/opmi_a_00147

**Published:** 2024-06-12

**Authors:** Aaron Chuey, Veronica Boyce, Anjie Cao, Michael C. Frank

**Affiliations:** Department of Psychology, Stanford University, Stanford, CA, USA

**Keywords:** methodology, meta-analysis, development, online studies

## Abstract

An increasing number of psychological experiments with children are being conducted using online platforms, in part due to the COVID-19 pandemic. Individual replications have compared the findings of particular experiments online and in-person, but the general effect of data collection method on data collected from children is still unknown. Therefore, the goal of the current meta-analysis is to estimate the average difference in effect size for developmental studies conducted online compared to the same studies conducted in-person. Our pre-registered analysis includes 211 effect sizes calculated from 30 papers with 3282 children, ranging in age from four months to six years. The estimated effect size for studies conducted online was slightly smaller than for their counterparts conducted in-person, a difference of *d* = −.05, but this difference was not significant, 95% CI = [−.17, .07]. We examined several potential moderators of the effect of online testing, including the role of dependent measure (looking vs verbal), online study method (moderated vs unmoderated), and age, but none of these were significant. The literature to date thus suggests—on average—small differences in results between in-person and online experimentation.

## INTRODUCTION

Developmental researchers are interested in studying children’s behavior, primarily by measuring their behavioral responses to experimental stimuli. Study sessions typically involve visits with local families in a laboratory setting or partnering with remote sites such as schools and museums. Although these interactions are a routine part of developmental research, they are time-consuming for both researchers and participants. Typical studies with dozens of infants or young children can require weeks or months of scheduling visits to a lab or many visits to testing sites. In-person testing also limits the participant pool to children living relatively close to the research site. Additionally, developmental research has been plagued by small, non-diverse samples even more so than research with adults due to limitations imposed by the demographics of the local population as well as the high costs of collecting data from children (Kidd & Garcia, 2022; Nielsen et al., 2017).

Prior to the rise of video chat software, there were only limited alternatives to in-person interaction for collecting experimental behavioral data from children. However, with the development of inexpensive and reliable video conferencing technology in the 2010s, new frontiers began to emerge for developmental testing.^1^ Indeed, even infants appear to follow others’ gaze (Capparini et al., 2023) and establish joint attention over video chat (McClure et al., 2018). Researchers soon experimented with conducting developmental studies through video-chat platforms, which in theory broaden the pool of participants to anyone at nearly any time and location so long as they have access to internet and an internet enabled device. What began as a few research teams experimenting with online studies (e.g., Lookit: Scott & Schulz, 2017; The Child Lab: Sheskin & Keil, 2018; Pandas: Rhodes et al., 2020) quickly expanded to much of the field as researchers scrambled to conduct safe research during the Covid-19 pandemic. This shift in research practices has yielded many empirical publications where some or all of the data were collected online. In addition, there is a growing literature on online methodology and best practices for designing such studies (we will not review this guidance here, but see e.g., Chuey, Asaba, et al., 2021; Kominsky, Begus, et al., 2021).

Some researchers may be eager to return to in-person testing, but online research is likely here to stay and may increase in frequency as communications technologies improve and become more accessible. Online testing has immense potential to change developmental science (Sheskin et al., 2020), much as crowdsourced testing of adults has changed adult behavioral science (Buhrmester et al., 2011). This potential has yet to be fully realized, however, as researchers have yet to fully understand the strengths and weaknesses of this method, as well as how to recruit diverse populations for online studies. Despite undersampling certain populations (Lourenco & Tasimi, 2020), online studies nonetheless allow researchers to sample from a larger, broader pool of participants than ever before as access to the internet continues to increase worldwide. Large, low cost samples and remote cross-cultural research may even become a reality for developmental researchers in the coming years.

How different are the results of developmental studies conducted online to those conducted in person? Direct comparison of effects measured in both modalities is critical to answering this question. Researchers have implemented a number of paradigms online and replicated their in-person findings, but it is still largely unknown how the strength of findings yielded from online developmental studies more broadly compares to those conducted in-person. Therefore, the current meta-analysis seeks to estimate effect sizes for phenomena measured with children online and for the same phenomena measured in closely-matched in-person studies. These study pairs in turn allow estimation of the average magnitude of the difference between in-person and online studies.

On the one hand, there is good reason to suspect that modality has little influence over the strength of a study’s effect. Fundamentally, studies conducted online and in-person utilize similar measures (e.g., looking time, verbal report) and use similar kinds of stimuli (e.g., moving objects, narrated vignettes). Additionally, experimenters still need to contend with extraneous factors like inattention, environmental distractions, and participants’ mood. On the other hand, meaningful differences in online and in-person interactions could affect the outcomes of online and in-person studies, in either direction.

In principle, researchers have more control over a child’s environment in-person, and in-person studies are usually less susceptible to technical problems such as lag or auditory or visual fidelity issues. Conversely, participants typically complete online studies in a more comfortable, familiar environment—their own home. Any of these factors could tip the scales, yielding larger effects in-person or online; as a result, we do not make any predictions regarding the presence or direction of an effect of study modality. Further, there are many ways in which online studies vary including whether a live experimenter is present, what the dependent measure is (e.g., preferential looking vs. reaching), and the age of the sample being tested. Such factors could also influence the outcomes of online and in-person studies.

Online studies are generally conducted in one of two formats: moderated or unmoderated. In moderated studies, a live experimenter guides participants through a study much like they would in-person, except online, typically via video-chat. Moderated studies are often operationalized as slides or videos shared with participants while the participants’ verbal responses or gaze is recorded. In contrast, with unmoderated studies participants complete a study without the guidance of a live experimenter. Instead, researchers create a preprogrammed module that participants or their caregivers initiate and complete according to instructions. Since no experimenter needs to be present and participants can participate at any time they choose, unmoderated studies offer the potential for fast, inexpensive data collection. However, since they lack an experimenter, participants’ experiences also deviate more from in-person studies compared to moderated studies that retain the same core social interaction between experimenter and participant. Therefore, it is possible that data collected via unmoderated sessions is comparatively noisier since an experimenter is unable to focus children’s attention or course correct like they can during a live interaction. We consider this possibility in the current meta-analysis.

Like developmental studies more broadly, online studies have also employed a number of dependent measures, including verbal and looking measures. Verbal measures are typically straightforward to record, while recording looking measures is more complex. Accurate looking measures require precise camera positioning and coding schemes, and are thus more likely to deviate from their in-person counterparts compared to studies that measure children’s verbal responses. To that end, automated gaze annotation is currently being developed and represents an exciting future direction in online methodology (Chouinard et al., 2019; see Erel et al., 2022; Steffan et al., 2024). We examine how the kind of dependent measure employed (looking vs. verbal) might moderate the difference between online and in-person results.

The final moderator we consider is participants’ age. Online developmental studies have sampled from a wide age range, including infants (e.g., Dillon et al., 2020), toddlers (e.g., Lo et al., 2021), preschoolers (e.g., Schidelko et al., 2021), and elementary schoolers (e.g., Chuey et al., 2020; Chuey, McCarthy, et al., 2021). Because online studies are often conducted in the comfort of their own homes, it is possible that children of all ages might benefit from this aspect of online studies. Conversely, because a child’s environment is more difficult to moderate online, infant studies, which often rely on precise environmental setups, may suffer more when conducted online. In addition, as children get older they may gain more experience with on-screen displays, which can contribute to their performance in online studies. We test these competing age moderation hypotheses.

In sum, to estimate the average effect size associated with online study administration for young children, we conducted a meta-analysis of matched studies conducted online and in-person; this includes online studies that replicated an older study conducted in-person as well as pairs of online and in-person studies conducted in parallel. In addition, we asked whether these differences are moderated by study format, dependent variable, or participant age.

We stress that our goal here is not to provide a conclusive, binary answer to the question of whether online and in-person studies are the same or different. Likely with enough studies to analyze, we would find that there are many cases when they are similar and some where they are different. Instead, our goal is to provide a best guess as to, on average, how different an effect would be if it was measured online vs. in-person. Even if there is some uncertainty in this estimate due to heterogeneity and the limited number of available comparative studies, we believe it is an important piece of information for developmental researchers as they plan the modality of their next study.

## METHODS

**Figure 1.**
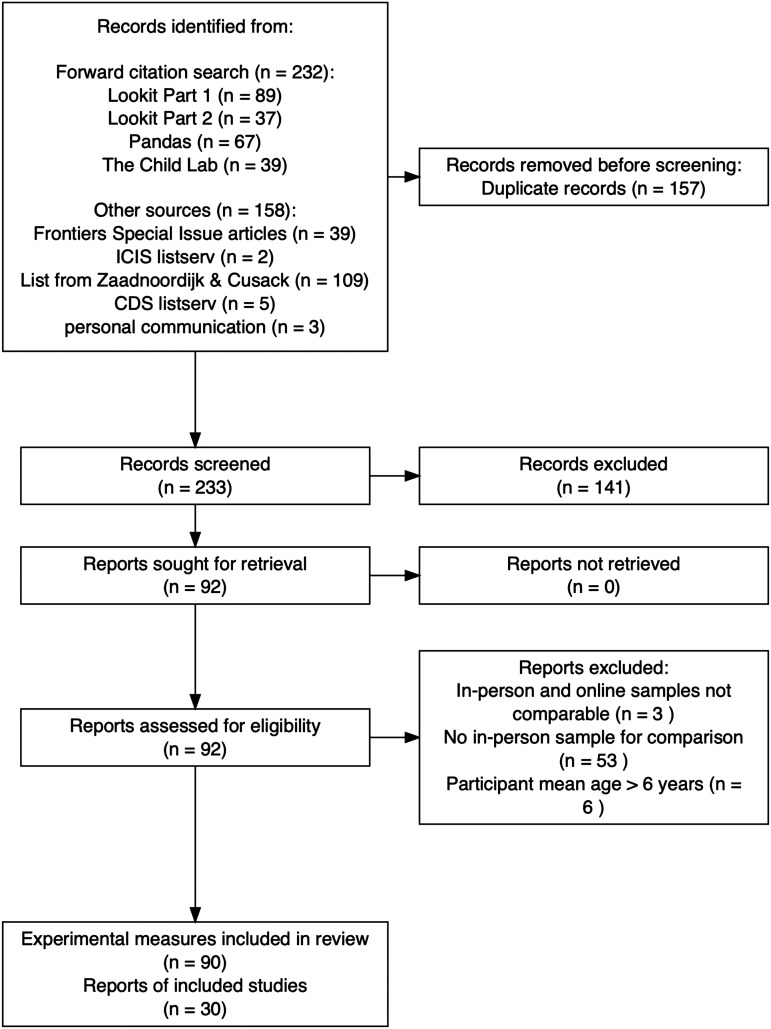
PRISMA plot detailing our study screening process; numerical values represent the number of papers at each stage of the systematic search.

We conducted a literature search following the Preferred Reporting Items for Systematic Reviews and Meta-Analyses (PRISMA) procedure (Page et al., 2021); see Figure 1. For each set of studies determined to be an online replication, we calculated the effect size(s) and associated variance for the main effect of interest. We then conducted a series of random-effects multilevel meta-regressions to estimate the effect of online data collection, as well as three possible moderators (online study method, type of dependent measure, and participant age). Our preregistered data selection, coding, and analysis plan can be found at https://osf.io/hjbxf. The list of papers included in this meta-analysis is shown in Table 1.

**Table 1.** Papers used in this meta-analysis, ordered by average participant age (in months). Some papers contained both online and in-person results, others contained online replications compared to previous in-person papers. Pairs refers to the number of paired online and in-person effect sizes contributed by each paper (set). Look is whether the studies use looking, verbal, or both types of dependent measures. Mod is whether the online studies were moderated, unmoderated, or both.

Paper	Pairs	Look	Mod	Age
Gasparini et al. (2022)	5	Verb	Mod	4
Bánki et al. (2022)	4	Look	Unmod	5
DeJesus et al. (2021)	3	Verb	Mod	5
McElwain et al. (2022)	27	Both	Mod	6
Bochynska and Dillon (2021) compared to Dillon et al. (2020)	2	Look	Unmod	7
Bulgarelli and Bergelson (2022)	3	Look	Mod	8
Yuen and Hamlin (2022) compared to Hamlin (2015)	2	Both	Mod	9
Beckner et al. (2023)	1	Look	Unmod	9
Smith-Flores et al. (2022) compared to Stahl and Feigenson (2015)	3	Look	Mod	13
Smith-Flores (2022) compared to Skerry and Spelke (2014)	2	Look	Mod	13
Lo et al. (2021)	1	Verb	Unmod	19
Margoni et al. (2018)	2	Look	Mod	21
Steffan et al. (2024)	1	Look	Mod	22
Nguyen et al. (2024)	2	Verb	Mod	22
Chuey, Asaba, et al. (2021)	3	Both	Mod	24
Mani (2022)	1	Look	Mod	24
Morini and Blair (2021)	1	Verb	Mod	30
Silver et al. (2021)	1	Verb	Mod	33
Schidelko et al. (2021)	4	Verb	Mod	44
Lapidow et al. (2021)	4	Verb	Both	44
Scott et al. (2017) compared to Téglás et al. (2007) and Pasquini et al. (2007)	17	Both	Unmod	45
Yoon and Frank (2019)	2	Verb	Unmod	48
Kominsky, Shafto, et al. (2021)	1	Verb	Mod	55
Escudero et al. (2021)	2	Verb	Mod	57
Vales et al. (2021)	3	Verb	Mod	58
Nelson et al. (2021)	8	Verb	Mod	59
Gerard (2022)	1	Verb	Unmod	60
Wang and Roberts (2023)	1	Verb	Mod	60
Aboody, Huey, et al. (2022)	1	Verb	Mod	60
Aboody, Yousif, et al. (2022)	1	Verb	Mod	72

### Literature Search

Our goal was to find as many published and unpublished online replications of developmental studies as possible. However, because there is no common nomenclature for online replications and the studies themselves cover a wide range of research questions and methodologies, searching via specific terms or keywords was difficult and produced many irrelevant papers; as a result, we could not conduct a completely systematic review. Instead, we preregistered a forward citation search strategy based on key papers on online developmental research. We used the papers that conducted initial validation of popular online testing platforms as our seeds, including Lookit (Scott et al., 2017; Scott & Schulz, 2017), The Child Lab (Sheskin & Keil, 2018), and Pandas (Rhodes et al., 2020). Any paper that cited at least one of these papers was considered for inclusion in our meta-analysis. We also considered all papers published in the Frontiers in Psychology Special Issue: Empirical Research at a Distance: New Methods for Developmental Science, which largely focused on online developmental studies and replications. Additionally, we were pointed to Zaadnoordijk and Cusack (2022) which contained a list of online replication papers, although this yielded few additional replications. Finally, we posted a call for contributions to the Cognitive Development Society (CDS) and International Congress of Infant Studies (ICIS) listservs, two popular emailing lists frequented by developmental researchers. This call yielded several publications our initial search strategy missed, as well as six unpublished but complete online replications.

We preregistered several eligibility criteria to filter articles from our search:

1. The study must be experimental, where participants complete a task with a stimulus. This criterion precludes surveys or purely observational measures.
2. The studies must report two groups of children, one tested online and another tested in-person. Although the online sample must be collected by the researchers reporting the results, the in-person sample could either be collected at the same time or referenced from an existing publication.
3. The mean age of the sample should be under six years. This criterion limits the studies to those conducted on relatively younger children for whom online data collection methods have not been traditionally employed.
4. All data reported or referred to must contain codable effect sizes. Verbal comparison alone between an online or in-person study or a qualitative description of results is not enough to determine the precise effect size of interest.
5. Data collection for both the in-person and online sample must be complete; any incomplete or partial samples were not considered. This criterion aimed to limit the inclusion of effect size estimates that might be biased by missing data, although in practice no datasets were excluded for being incomplete.
6. The online and in-person methods must be directly comparable. Some alteration to the study methods is expected when adapting an in-person study to be run online (e.g., having children refer to objects by color instead of pointing). However, we excluded any studies whose methodologies altered the nature of the task or the conclusions that could be drawn from them (e.g., changing the identity of a hidden object instead of its location in a false belief task).

### Data Entry

All papers (233) yielded by our search procedure went through three rounds of evaluation to determine if they met our inclusion criteria. First, we screened the titles of the papers to determine whether they might include an online experiment. Those that clearly did not meet one or more of our inclusion criteria were excluded from further evaluation. Next, we performed a similar evaluation based on the papers’ abstracts, before a final round based on the article as a whole. All remaining papers were entered into a spreadsheet that coded the necessary information for calculating the size of the main effect(s) of interest and their associated variance (sample size, group means and standard deviation, and t and F statistics when applicable), as well as our preregistered moderators (study modality, data collection method, dependent measure, and participant age).

If a paper reported an effect size as Cohen’s *d* (referred to below as standardized mean difference, SMD), we coded it directly. Otherwise, we calculated the individual effect sizes for each main effect and each study (online and in-person) via reported means and standard deviations, t-statistic, or directly from the data if it was available using analysis scripts adapted from Metalab (e.g., Bergmann et al., 2018), a repository of meta-analyses in early language and cognitive development. If the main comparison was to chance performance, we first calculated log odds and then converted the effect size to Cohen’s *d* via the compute.es package in R (Del Re & Del Re, 2012). If a given study had multiple dependent measures or central hypotheses, we calculated an effect size and associated variance for each.

### Analytic Approach

To determine whether study modality (online or in-person) moderated the size of the main effect of interest for each set of studies, we performed a preregistered random-effects multilevel meta-regression using the metafor package in R (Viechtbauer, 2010). The regression predicted individual study effect size (SMD) with study modality as a fixed effect, modeling individual experimental effect sizes with the coefficient of interest being the study modality predictor (online vs. in-person). As discussed above, we did not predict a direction of effect for the study modality predictor.

Our approach focused on the study modality moderator, rather than computing an online-offline difference score for each study and estimating the size of that difference directly. Although at a first glance this approach may seem simpler, many papers are heterogeneous and contain multiple online studies for a single given offline study, or multiple measures within the same study. In these cases, the appropriate difference was not always clear. For this reason, we chose to enter all study effects into the meta-regression and use the study modality moderator to estimate systematic modality effects.

To ensure that differences in the total number of effect sizes across studies did not bias our analysis by over-weighting studies with more measurements, we used a four-level nested random effects structure, assigning random intercepts to each hierarchical level. Individual papers (level 1) contain sets of experiment pairs (level 2) that involve one or more samples of participants (level 3) from which individual effect sizes are derived (level 4). In effect, level 1 captures variation between different papers or pairs of papers included in our meta-analysis, level 2 captures variation between particular experiments within those papers (e.g., modeling the dependency between multiple measurements reported from a single experiment), level 3 captures variation between different groups of participants (e.g., modeling the dependency between effect sizes from participants who completed a battery of tasks with multiple effects of interest), and level 4 captures variation between different effect size measures within a single sample (e.g., modeling the dependency between multiple effect sizes yielded by a particular sample from a particular experiment). There are three instances in our sample where the same group of participants participated in multiple different experiments, breaking this dependency structure. To our knowledge, it is not currently possible to fit crossed random effects structures, however, and because this was a rare exception, we retained the four-level nested structure. We had originally preregistered a simpler random effects structure that assigned random intercepts by experiment and sample independently. However, this strategy incorrectly assumed these factors were independent, so we modified our models as described.

To determine the effect of additional moderators—online study method (moderated vs unmoderated), dependent measure (looking vs verbal), and participant age—we conducted three additional multilevel meta-regressions each with an additional fixed effect plus the corresponding interaction with study modality. All analysis scripts were preregistered (except the random effects structure, as above), and the code is available at https://osf.io/up6qn.

## RESULTS

### Planned Analysis

**Figure 2.**
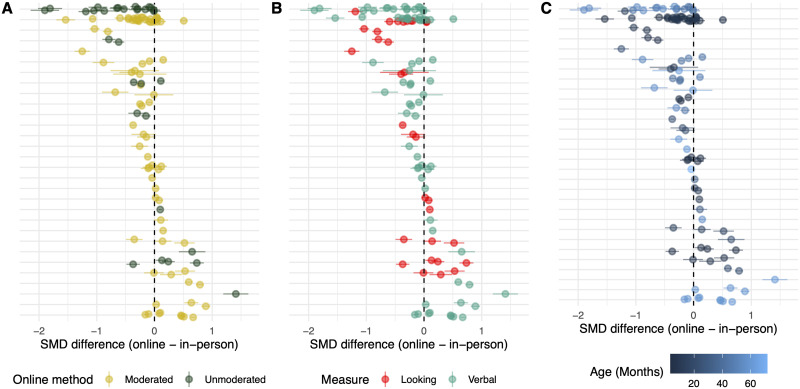
Forest plots of studies, sorted by difference in SMD. Each row is one study (paper or pair of papers) and contains every effect size pair contributed by that study. Each dot represents the difference between a single in-person measure and a corresponding online measure. A: Difference in SMD by study and online method (moderated vs unmoderated). B: Difference in SMD by study and measurement type (looking vs verbal). C: Difference in SMD by study and mean participant age (months).

Overall, the meta-analysis revealed a small negative, non-significant effect of online study modality, Est = −0.05, 95% CI = [−0.17, 0.07], *p* = 0.455. Additionally, we did not find any significant effect of our preregistered moderators or any significant interactions between the moderators and study modality. See Table 2 for coefficient values. Figure 2 shows the effect size differences of experiments by moderators.

**Table 2.** Mean SMD across studies by study modality, data-collection method, and type of dependent measure.

Modality	Method	Measure	*N* (Effect-size Pairs)	SMD	95% CI
In-person	Moderated	Looking	14	0.797	[0.476, 1.119]
In-person	Moderated	Verbal	36	0.509	[0.325, 0.694]
Online	Moderated	Looking	12	0.573	[0.264, 0.882]
Online	Moderated	Verbal	30	0.439	[0.294, 0.585]
Online	Unmoderated	Looking	5	0.214	[0.062, 0.367]
Online	Unmoderated	Verbal	5	1.311	[0.193, 2.429]

Because our meta-analysis averaged across effects from very different paradigms (which could yield different effect sizes independent of the effect of testing modality), we expected substantial heterogeneity. Consistent with that expectation, all tests for residual heterogeneity were highly significant (all *p*s < .0001). Values of *σ*^2^ (the mean random effect variance of the model for each nested level, from level 1–4) for all models were 0.187, 0, 0.051, 0.018 (primary model), 0.193, 0, 0.049, 0.018 (moderated vs. unmoderated model), 0.188, 0, 0.049, 0.019 (looking-time model), and 0.197, 0, 0.048, 0.017 (age model), respectively, confirming the impression that these moderators did not reduce heterogeneity.

### Exploratory Analysis

In addition to our multi-level meta-analysis, we examined which combinations of methods and measures tended to yield the strongest and weakest effect sizes relative to their in-person counterparts. We fit a meta-analytic model containing method, response mode, and modality as well as their two- and three-way interactions, with the same random effects structure as our previous model. We cannot draw any strong conclusions about these noisy estimates due to our relatively small sample size. That said, descriptively, unmoderated online studies with looking measures were estimated to have noticeably smaller effect sizes compared to both their moderated online and in-person counterparts (see Table 2). In contrast, as estimated by this model, moderated online studies with looking and verbal measures as well as unmoderated online studies with verbal measures did not show such large differences from their in-person counterparts.

We also conducted an exploratory analysis of potential publication bias. It was unclear *a priori* how we might expect publication biases to manifest themselves, given that there is some possibility of notoriety for either showing *or* failing to show differences between online and in-person testing. In either case our hypothesized selection process operated on the *differences* in effect sizes between each online and in-lab pair of samples.

**Figure 3.**
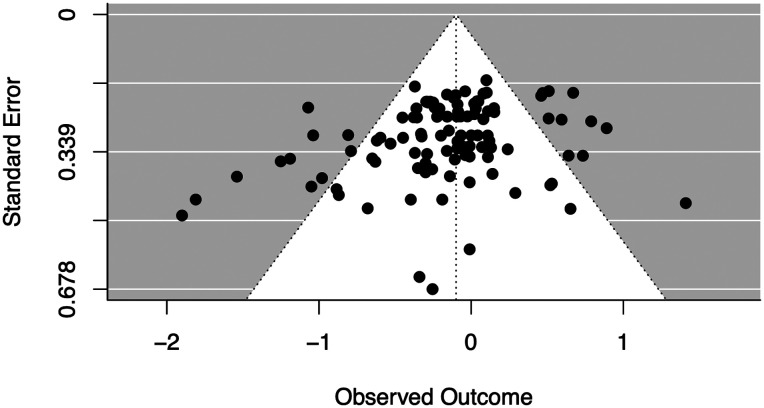
Funnel plot of the differences in effect size between pairs of in-person and online studies. A positive observed outcome means the online study had a larger effect size.

For each online and in-person pair on the same study, we calculated a standard mean difference in effect size between the two studies as well as the variance of this difference. The resulting funnel plot is shown in Figure 3. As the difference in effect size increases, the variance should also increase; however, if asymmetries are observed in this relationship (e.g., a greater number of negative outcome values with low variance), effect sizes may not have been uniformly reported. According to Egger’s regression test for funnel plot asymmetry, a common method for assessing publication bias in meta-analyses, this plot is asymmetric (*p* = .005) and the estimated effect assuming no variance is 0.26 [−0.03, 0.55]. This analysis suggests the possibility of publication bias favoring studies that have smaller effect sizes online compared to in-person, signaling that perhaps online studies may have relatively larger effect sizes on average compared to what has been reported. We interpret this conclusion with caution, however, noting the large width of the estimated CI and the relatively low power of Egger’s test (Sterne et al., 2000).

## DISCUSSION

The current meta-analysis provides a birds-eye view of how developmental studies conducted online compare with closely matched counterparts conducted in-person. Our results suggest that overall, comparable studies yield relatively similar effect sizes. Even the upper end of the confidence interval for the online-offline difference estimate is still relatively small. This finding should be heartening for developmentalists interested in using online data collection.

We also examined whether modality effects emerged more substantially in particular settings, but did not find evidence for other moderators. The method of online data collection, type of dependent measure, and participant age did not have a significant impact on the effect of modality. Nonetheless, the lack of statistical precision, indicated by relatively wide confidence intervals, limits our ability to draw strong conclusions about the effect of any of our moderators. Future analysis is needed to determine the moderating effect, if any, that these factors exercise on the outcome of developmental studies conducted online.

The current analysis is coarse-grained, considering only one particular dichotomy within study modality: in-person vs online. Yet, there are many ways that developmental studies can be further subdivided. For example, studies are conducted both in quiet spaces (e.g., in lab, at home) and loud spaces (e.g., parks, museums), although the lack of granularity with respect to how these factors are reported in the literature renders us unable to examine them in the current meta-analysis. Therefore, online studies might over- or under-perform relative to studies conducted in particular in-person locations. Our moderators are also correspondingly course-grained, particularly dependent measure (looking vs verbal). Because our small sample size renders our analysis underpowered to detect weaker effects of moderators, the current results and their interpretation are subject to change as online methods improve and comparisons to in-person studies are better understood.

Unmoderated studies with looking measures had the noticeably smallest effect sizes relative to their in-person counterparts. This could reflect the difficulty of both collecting and coding looking data online using participants’ own webcams without significant real-time instruction. Indeed, it is possible that effect sizes suffer without a live experimenter eliciting and sustaining infants’ attention or guiding parents as they position and orient their infant. However, smaller effect sizes online could instead reflect genuinely smaller effect sizes of the underlying effect rather than a lack of online studies’ sensitivity. Developmental research has suffered from many failures to replicate in the past, especially studies with infants (e.g., Davis-Kean & Ellis, 2019), and many of the online studies in our sample were conducted after their in-person counterparts, sometimes years later. Therefore, it is possible that smaller online effect sizes simply represent a more accurate estimation of the true (smaller) effect rather than an effect of study modaility per se.

Unfortunately, the studies in our sample did not consistently report demographic information at the level of detail necessary for investigating how participants’ home environment socioeconomic status, or identity moderated the effect of study modality. Although children’s demographic characteristics influence their study performance no matter the modality, these factors arguably stand to exert a greater influence over the outcome of online studies because in-person studies standardize the study environment across participants while online studies do not. Thus, the effect sizes of studies conducted online are additionally at the mercy of participants’ home environment, and by extension the demographic factors that shape its features.

The composition of our sample might also bias our results. To match online and in-person methods as closely as possible, we only considered direct online replications for the current meta-analysis. While this approach ensures that data were collected online and in-person using similar methods and procedures, it limits our sample size and may bias our sample. For example, perhaps researchers disproportionately choose to conduct online replications of strong or well-established effects rather than replicate more subtle, weaker effects. Nonetheless, our analysis found that if publication bias exists, it likely favors stronger in-person effect sizes or non-replications among the studies we sampled. We also included an open call for unpublished data in an attempt to limit the file drawer problem (see Rosenthal, 1979).

Although developmental researchers have had decades of experience designing and running experiments in-person, most have only had a few years or less of experience developing online studies. Thus, our meta-analysis might also underestimate the potential of online research due to researcher and experimenter inexperience. Over the next several years, as developmental researchers develop expertise and experience with conducting research online, online studies might become more accurate at capturing cognitive constructs for any number of reasons, including better experimenter-participant interactions, better stimulus design (see Chuey, Asaba, et al., 2021), and more accurate methods of measurement (i.e., automatic looking time measures, see Erel et al., 2022). Relatedly, as new methods are developed and adapted for online experiments, researchers should not take the current findings as a blanket declaration that all online studies produce comparable results to their in-person counterparts; some might underperform, while others might outperform. Nonetheless, the current results suggest that across currently employed developmental methodologies, the effect sizes of studies conducted with children online are generally comparable to those conducted in-person, especially for studies utilizing verbal measures.

## CONCLUSION

Our meta-analysis found that, across closely matched developmental studies conducted in-person and online, the size of the main effect of interest for in-person studies was similar to the effect for online studies, yielding only a small average difference between them. While our sample of studies limits the precision of our estimates, nevertheless the general similarity in outcomes for in-person and online studies with children paint an optimistic picture for online developmental research more broadly going forward.

## ACKNOWLEDGMENTS

We thank everyone who contributed data to this meta-analysis.

## FUNDING INFORMATION

We don’t have any funding information to report given the low-cost nature of this work.

## AUTHOR CONTRIBUTIONS

A.C.: Conceptualization; Data curation; Formal analysis; Methodology; Visualization; Writing – original draft. V.B.: Conceptualization; Data curation; Formal analysis; Methodology; Visualization; Writing – review & editing. A.C.: Conceptualization; Data curation; Formal analysis; Methodology; Visualization; Writing – review & editing. M.C.F.: Conceptualization; Data curation; Formal analysis; Methodology; Supervision; Visualization; Writing – review & editing.

## DATA AVAILABILITY STATEMENT

All data and code referenced in this paper are available at https://osf.io/up6qn/.

## Note

^1^ Observational and survey research has long been conducted through the phone or by mail (e.g., Fenson et al., 1994); here we focus primarily on behavioral observation and experimental methods.
